# Differential Ecosystem Function Stability of Ammonia-Oxidizing Archaea and Bacteria following Short-Term Environmental Perturbation

**DOI:** 10.1128/mSystems.00309-20

**Published:** 2020-06-16

**Authors:** Jun Zhao, Yiyu Meng, Julia Drewer, Ute M. Skiba, James I. Prosser, Cécile Gubry-Rangin

**Affiliations:** a School of Biological Sciences, University of Aberdeen, Aberdeen, United Kingdom; b Centre for Ecology and Hydrology, Penicuik, United Kingdom; University of Waterloo

**Keywords:** land-use change, oil palm soil, pH perturbation, stability, tropical forest soil

## Abstract

Rapidly expanding conversion of tropical forests to oil palm plantations in Southeast Asia leads to soil acidification following intensive nitrogen fertilization. Changes in soil pH are predicted to have an impact on archaeal ammonia-oxidizing archaea (AOA), ammonia-oxidizing bacteria (AOB), and complete (comammox) ammonia oxidizers and, consequently, on nitrification. It is therefore critical to determine whether the predicted effects of pH on ammonia oxidizers and nitrification activity apply in tropical soils subjected to various degrees of anthropogenic activity. This was investigated by experimental manipulation of pH in soil microcosms from a land-use gradient (forest, riparian, and oil palm soils). The nitrification rate was greater in forest soils with native neutral pH than in converted acidic oil palm soils. Ammonia oxidizer activity decreased following acidification of the forest soils but increased after liming of the oil palm soils, leading to a trend of a reversed net nitrification rate after pH modification. AOA and AOB nitrification activity was dependent on pH, but AOB were more sensitive to pH modification than AOA, which demonstrates a greater stability of AOA than AOB under conditions of short-term perturbation. In addition, these results predict AOB to be a good bioindicator of nitrification response following pH perturbation during land-use conversion. AOB and/or comammox species were active in all soils along the land-use gradient, even, unexpectedly, under acidic conditions, suggesting their adaptation to native acidic or acidified soils. The present study therefore provided evidence for limited stability of soil ammonia oxidizer activity following intensive anthropogenic activities, which likely aggravates the vulnerability of nitrogen cycle processes to environmental disturbance.

**IMPORTANCE** Physiological and ecological studies have provided evidence for pH-driven niche specialization of ammonia oxidizers in terrestrial ecosystems. However, the functional stability of ammonia oxidizers following pH change has not been investigated, despite its importance in understanding the maintenance of ecosystem processes following environmental perturbation. This is particularly true after anthropogenic perturbation, such as the conversion of tropical forest to oil palm plantations. This study demonstrated a great impact of land-use conversion on nitrification, which is linked to changes in soil pH due to common agricultural practices (intensive fertilization). In addition, the different communities of ammonia oxidizers were differently affected by short-term pH perturbations, with implications for future land-use conversions but also for increased knowledge of associated global nitrous oxide emissions and current climate change concerns.

## Abstract (precis)

Physiological and ecological studies have provided evidence for pH-driven niche specialization of ammonia oxidizers in terrestrial ecosystems. However, the functional stability of ammonia oxidizers following pH change has not been investigated, despite its importance in understanding the maintenance of ecosystem processes following environmental perturbation. This is particularly true after anthropogenic perturbation, such as the conversion of tropical forest to oil palm plantations. This study demonstrated a great impact of land-use conversion on nitrification, which is linked to changes in soil pH due to common agricultural practices (intensive fertilization). In addition, the different communities of ammonia oxidizers were differently affected by short-term pH perturbations, with implications for future land-use conversions but also for increased knowledge of associated global nitrous oxide emissions and current climate change concerns.

## INTRODUCTION

The conversion of forests to oil palm plantations in Southeast Asia has rapidly expanded in recent decades due to its high profitability (1–3). Global oil palm cultivation increased from 3.6 to 19 million ha during the period 1961 to 2018, 63% of which is in Malaysia and Indonesia (FAO, 2018; http://www.fao.org/faostat/en/#data/QC). This land-use conversion process is marked by intense anthropogenic disturbance, including land clearing, soil drainage, road/track building, seedling plantations, and follow-up agricultural management (3), which impose a major threat to the biodiversity of native tropical forests (4–6). In particular, amendment with high levels of ammonium-based, mineral fertilizers is a common oil palm agricultural practice. The uptake of ammonium by plant roots is accompanied by proton release into the soil solution leading to significant decreases in soil pH (7, 8) that are likely to influence microbial community structure and activity (9). As tropical forests represent a crucial ecosystem for global carbon (C) and nitrogen (N) cycles (10–13), a better understanding of biogeochemical processes in these environments is required. The aim of this study was therefore to understand the impact of soil acidification associated with oil palm land conversion on the activity and the diversity of microbes involved in one of the key processes of the nitrogen cycle, nitrification.

Ammonia oxidation, the first step of nitrification (oxidation of ammonia via nitrite to nitrate), is a central process in the terrestrial nitrogen cycle. It is performed by ammonia-oxidizing archaea (AOA), canonical ammonia-oxidizing bacteria (AOB), and complete ammonia oxidizers (comammox), and several environmental factors have been invoked to explain their niche differentiation and specialization in terrestrial ecosystems. Of these, pH is particularly important in the ecology and evolution of ammonia oxidizers, controlling niche specialization of both archaeal and bacterial soil ammonia oxidizers (14–17). AOA generally dominate ammonia oxidation in acidic soils (18–20) and either AOA or AOB may dominate ammonia oxidation in slightly acidic or neutral pH soils (21–23), while the environmental distribution of recently discovered comammox in soils of different pH is not yet known. Although nitrification rates are traditionally considered to be lower in acid soils, due to reduced ammonia availability through ionization of ammonia to ammonium (24), the net nitrification rate does not show a strong relationship with soil pH (25), and this can be explained by the distribution and activities of physiologically diverse groups of AOA and AOB across a range of pH and ecosystems (15, 26, 27). There is also evidence that AOA and AOB have preferences for different sources of ammonia, with AOA favoring supply through mineralization of organic N, while AOB benefit from supply of high levels of inorganic N (28–31). These findings lead to contrasting hypotheses concerning the impact of oil palm land conversion on ammonia oxidizer communities, with AOB favored by high levels of inorganic N fertilization, while AOA benefit from soil acidification. Functional redundancy across AO communities may reduce impacts on ammonia oxidation rates, which may be similar across land-use gradients (forest to oil palm), especially in established ecosystems in which microbial communities have had sufficient time to adapt following perturbation (e.g., in well-established forests or older palm oil conversions).

Ecosystem process stability following an environmental disturbance depends strongly on recovery and/or adaptation of microbial communities to new conditions (32, 33). Resistance and resilience and the consequent stability of ammonia oxidizers following disturbance have rarely been studied, but there is some evidence that AOB populations are more resistant and resilient than AOA populations to drying-rewetting events in nonadapted soils (34), probably through lower sensitivity of AOB than AOA to water stress (both matric and osmotic potential) (35). However, little is known of the stability of ammonia oxidizer communities following changes in soil pH, despite its ecological importance. pH niche specialization of AOA and AOB suggests that AOA communities are active under a wider range of soil pH than AOB, while evidence for growth of acid-adapted AOB remains scarce (24, 36, 37). Therefore, one can presume that AOA populations are more stable than AOB following a decrease in pH, with subsequent nitrification activity being driven mainly by AOA, especially in recently modified land.

This study therefore aimed to test the hypothesis that land-use conversion (forest to oil palm) does not affect nitrification rate, due to high functional redundancy of AO communities, especially in well-established tropical ecosystems. In addition, it is proposed that AOA populations have greater stability than AOB populations in response to pH change, which is an impact of such land-use conversion. These hypotheses were tested by measuring both nitrification rate and the activity and stability of AOA and AOB communities following soil pH change, using microcosms containing several soils from a natural ecosystem gradient in Sabah, Malaysian Borneo.

## RESULTS

### Net nitrification rates in different land-use soils and following pH perturbation.

**FIG 1:**
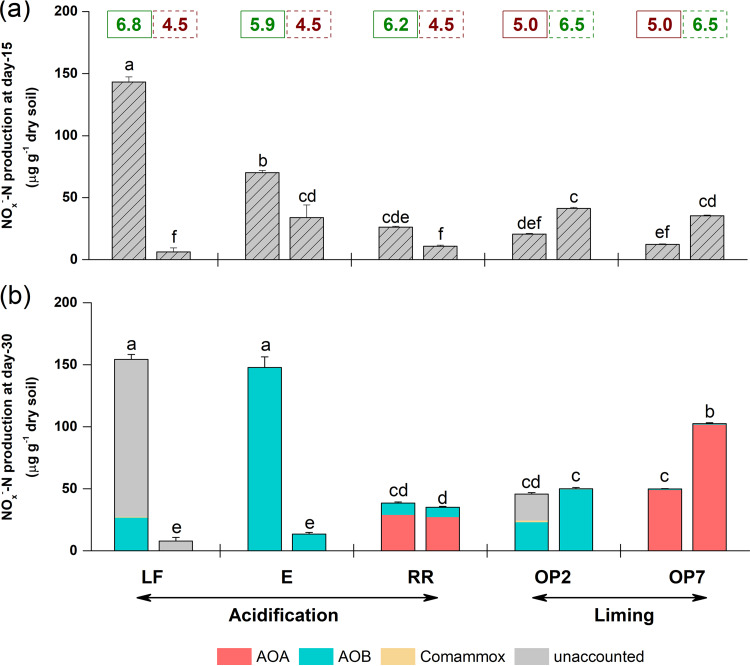
Temporal changes in nitrite plus nitrate (NO_x_^−^) concentration following incubation of microcosms for 15 (a) and 30 (b) days and putative contributions of ammonia oxidizers after 30 days (b). Microcosms were constructed using a gradient of land usage: two forest soils (LF and E), a riparian soil (RR), and 2- and 7-year-old oil palm soils (OP2 and OP7). NO_x_^−^ production was calculated as the difference in NO_x_^−^ concentration between day 0 and day 15 or 30 at native or modified pH, with the number above each column referring to soil pH (green and red numbers represent the high-pH and low-pH values, respectively, while the solid and dotted line boxes represent native-pH and modified-pH values, respectively). The contributions of AOA, AOB, and comammox to NO_x_^−^ production after 30 days were estimated as the number of cells assimilating CO_2_ (estimated by the number of cells in the heavy fractions of the ^13^CO_2_-labeled microcosms) multiplied by their recorded highest maximum specific cell activity (2.6 fmol NH_3_ cell^−1^ h^−1^ for AOA, 23 fmol NH_3_ cell^−1^ h^−1^ for AOB and 2.6 fmol NH_3_ cell^−1^ h^−1^ for comammox, respectively). The sum of these three absolute NO_x_^−^ production estimates resulted in some cases in a lower theoretical value than the NO_x_^−^ production value measured: hence the assignment of “unaccounted” contribution. Triplicate day 0 and six day 15 and six day 30 microcosms (triplicate ^12^CO_2_-amended and triplicate ^13^CO_2_-amended microcosms) were sampled to calculate mean values, and the error bars represent standard errors. Different letters above the bars in each panel indicate significant differences in the levels of NO_x_^−^ production.

Net nitrification rates were estimated as temporal changes in NO_x_^−^ concentrations and were positive in all soils (Fig. 1). Nitrification led to a significant decrease in pH in all except the acidified soils, for which pH increased slightly (*P < *0.05), presumably due to soil buffering (see Fig. S1 in the supplemental material).

10.1128/mSystems.00309-20.1 FIG S1 Temporal changes in soil pH and ammonium concentration in microcosm-containing soil at native pH and modified pH. Three native neutral soils (LF, E, and RR) were acidified, and two native acidic soils (OP2 and OP7) were limed. Triplicate day 0, six day 15, and six day 30 microcosms (triplicate [^12^C]CO_2_-amended and triplicate [^13^C]CO_2_-amended microcosms) were sampled to calculate mean values, and the error bars represent standard errors. The crosshatch symbol (#) indicates a significant temporal change in the measured parameter for each set of soil/pH incubation conditions. The black asterisk (*) indicates a significant difference between the high-pH and low-pH conditions at a specific time point within each soil. Download FIG S1, TIF file, 0.07 MB. Copyright © 2020 Zhao et al. 2020 Zhao et al. This content is distributed under the terms of the Creative Commons Attribution 4.0 International license.

Both soil type (gradient of forest to oil palm) and pH perturbation (acidification or liming) significantly influenced the net nitrification rate (*P < *0.001). At native pH, the nitrification rate was significantly higher in forest soils (LF and E) than in riparian (RR) and oil palm soils (OP2 and OP7) (Fig. 1). Following pH perturbation, soil acidification decreased the nitrification rate in the forest soils after both 15 and 30 days and this decrease was not due to a low mineralization rate, as the ammonium concentration was sufficiently high and accumulated during incubation of both forest soils following acidification (Fig. S1). Soil acidification led to a significantly lower nitrification rate, after incubation for 15 days, in the pH-modified soil than in the native riparian soil (Fig. 1a), but rates in soils were similar after incubation for 30 days (Fig. 1b). However, the concentration of ammonium in both native-pH and modified-pH riparian soils was low and potentially limiting after 15 days (Fig. S1), possibly due to a low mineralization rate in this soil. Soil liming increased the nitrification rate in the long-established oil palm soil (OP7) after both 15 and 30 days and in the younger oil palm soil (OP2) after 15 days but not after 30 days (Fig. 1). Again, these different responses to pH perturbation after 15 and 30 days were likely due to ammonium limitation in the second period of incubation (Fig. S1). Following incubation for 30 days, pH modification inverted the net nitrification rate along the land-use gradient (riparian and oil palm soils > forest soils, Fig. 1b).

### Ammonia oxidizer abundance and stability in response to pH perturbation.

**FIG 2:**
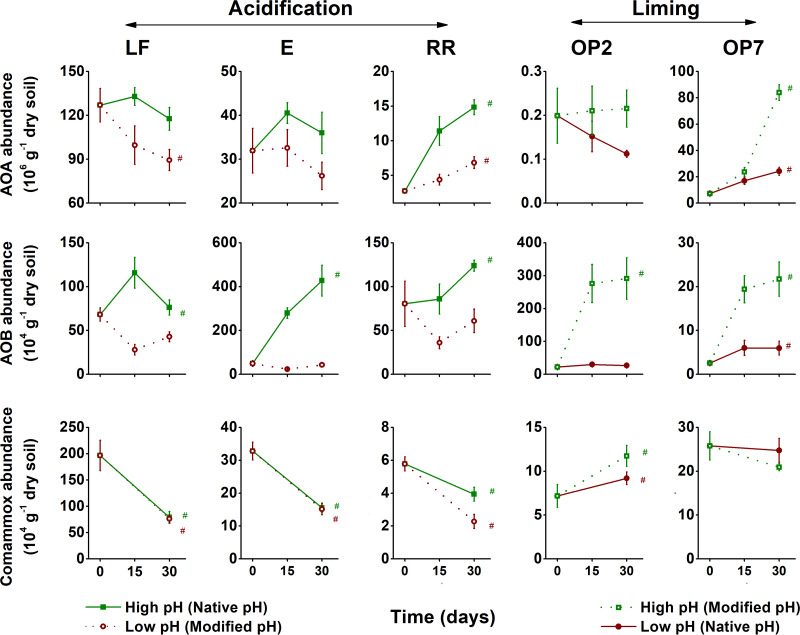
Temporal changes in archaeal (AOA), bacterial (AOB), and complete (comammox) ammonia oxidizer abundances in microcosms containing soils from the land-use gradient, consisting of two forest soils (LF and E), a riparian soil (RR), and 2- and 7-year-old oil palm soils (OP2 and OP7), at native pH and after changes in pH. Triplicate day 0, six day 15, and six day 30 microcosms (triplicate ^12^CO_2_-amended and triplicate ^13^CO_2_-amended microcosms) were sampled to calculate mean values, and the error bars represent standard errors. The number sign (#) indicates a significant temporal change (increase or decrease) in the measured abundance for each set of soil/pH incubation conditions (*P < *0.05).

**FIG 3:**
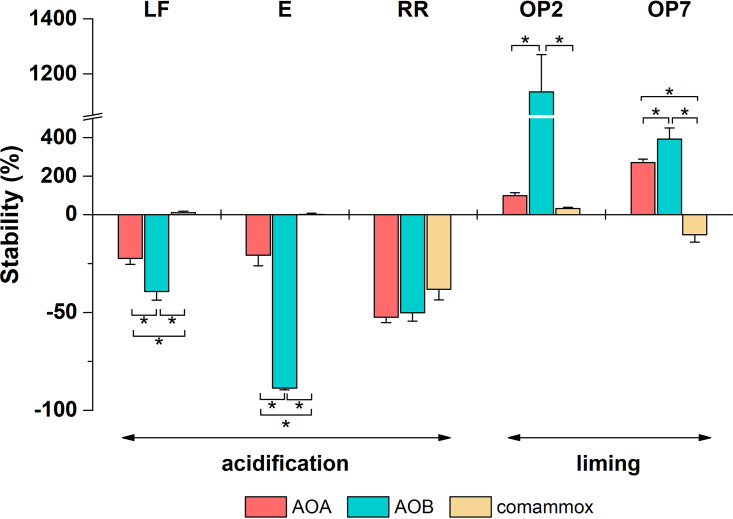
The stability of AOA, AOB, and comammox abundance following pH modification (acidification or liming) and incubation for 30 days of the five soils from the land-use gradient: two forest soils (LF and E), a riparian soil (RR), and 2- and 7-year-old oil palm soils (OP2 and OP7). Stability was estimated as the proportional change in archaeal, bacterial, or comammox *amoA* gene abundances in modified-pH (*M*) soils compared to native-pH (*N*) soils at day 30, using the following equation: stability = [(*M* − *N*)/*N*] × 100. For each community, the highest stability is achieved at the neutral point (stability = 0), while deviation from the neutral point indicates overcompensation (positive value) or undercompensation (negative value) mechanisms, representing a relative increase or decrease in the pH-perturbed environment compared to the native environment, respectively. An asterisk above or below a pair of bars indicates a significant difference between AOA, AOB, and comammox stability in the corresponding soil (*P < *0.05).

AOA, AOB, and comammox were each detected in the five soils, and their abundances were affected 30 days after soil pH perturbation in different ways (Fig. 2). The abundances of AOA and AOB ranged from 1.1 × 10^5^ to 1.3 × 10^8^ and from 2.5 × 10^4^ to 4.3 × 10^6^ g^−1 ^dry soil in different soils, respectively, and were higher under higher-pH conditions in each soil after incubation for 30 days (Fig. 2). Comammox abundance ranged from 2.3 × 10^4^ to 2.0 × 10^6^ g^−1 ^dry soil in different soils but showed no consistent pattern with high-pH and low-pH conditions (Fig. 2). Specifically, after incubation for 30 days, soil acidification led to significant decreases in AOA and AOB abundances of 18% to 57% and 39% to 91%, respectively, in all three acidified soils (LF, E, and RR) in comparison to the native-pH soils (Fig. 3). In contrast, liming significantly increased AOA and AOB abundances, by 62% to 270% and 381% to 1,134%, respectively, in the two limed soils (OP2 and OP7) (Fig. 3). Interestingly, similar pH perturbations had different effects on comammox abundance in different soils. For instance, soil acidification significantly decreased comammox abundance in the riparian soil but did not change comammox abundance in the two forest soils (Fig. 3). In addition, liming led to contrasting effects on comammox abundance in the young and old oil palm soils (Fig. 3).

In all soils (except the riparian soil), pH perturbation altered the abundance of AOB to a greater extent than that of AOA or comammox (*P < *0.05) (Fig. 3), suggesting that AOA and comammox were less affected than AOB by pH perturbation. The stabilities of AOA, AOB, and comammox communities in response to pH perturbation were similar in the riparian soil (Fig. 3), which has been subjected to the least anthropogenic activity, and these ammonia oxidizer communities suffered from undercompensation mechanisms, suggested by their negative stability following pH perturbation.

### Ammonia oxidizer growth and putative contribution to nitrification.

Ammonia oxidizer growth was first estimated as the temporal increase in *amoA* gene abundance during incubation for 30 days. This approach demonstrated growth of AOA in the riparian (RR) and old oil palm (OP7) soils, irrespective of pH, and growth of AOB in all soils at higher pH and in acidic OP7 soil (Fig. 2). Comammox growth was detected only in young oil palm soil OP2 at both low and high pH (Fig. 2).

**FIG 4:**
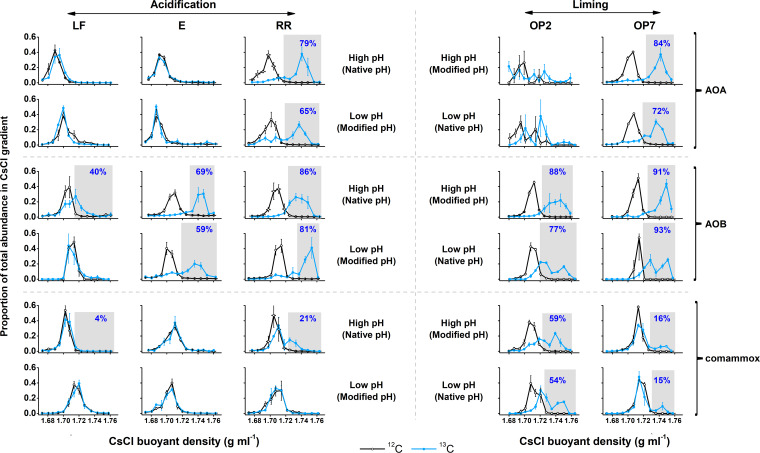
Buoyant density distributions of archaeal (AOA), bacterial (AOB), and complete (comammox) ammonia oxidizer abundance after incubation of microcosms for 30 days with [^12^C]CO_2_ or [^13^C]CO_2_. Microcosms were constructed using a gradient of land usage: two forest soils (LF and E), a riparian soil (RR), and 2- and 7-year-old oil palm soils (OP2 and OP7). The plotted values are the relative abundances of AOA, AOB, or comammox *amoA* genes in each fraction as a proportion of the total abundance across the whole CsCl gradient. Vertical error bars represent standard errors of relative abundances from triplicate microcosms, and the horizontal error bars represent standard errors of buoyant density of the same order fraction from six microcosms (triplicate [^12^C]CO_2_ and triplicate [^13^C]CO_2_ treatments).

DNA stable-isotope probing (DNA-SIP) was further used to assess growth of autotrophic ammonia oxidizers (through assimilation of [^13^C]CO_2_ into *amoA* genes), and autotrophic growth of AOA, AOB, and/or comammox was observed in all incubated soils except LF soil following acidification (Fig. 4). Approximately 65% to 84% of AOA, 40% to 93% of AOB, and 4% to 59% of comammox species were labeled with ^13^C in different soils following microcosm incubation, and DNA-SIP confirmed all ammonia oxidizer growth observed by a temporal increase in *amoA* gene abundance (by quantitative PCR [qPCR]). DNA-SIP results were consistent with the assessment of AOA growth by qPCR (Fig. S2), and also allowed determination of bacterial ammonia oxidizer growth that was not detected by temporal increases in *amoA* gene abundance, with additional detection of AOB and comammox growth in four and three different soils, respectively (LF, E, RR, and OP2 for AOB and LF, RR, and OP7 for comammox) (Fig. 4; see also Table 1).

**TABLE 1:** Abundances of growing ammonia oxidizers in each native or modified-pH soil of the land usage gradient, including two forest soils (LF and E), a riparian soil (RR), and 2- and 7-year-old oil palm soils (OP2 and OP7)^*a*^

Soil and category	Abundance of growing ammonia oxidizers (no. of *amoA* genes g^−1 ^dry soil) determined by indicated assay					
	AOA growth		AOB growth		Comammox growth	
	qPCR	SIP	qPCR	SIP	qPCR	SIP
LF						
High pH (native pH)	n.d.	n.d.	n.d.	2.9 × 10^5^ ± 6.9 × 10^4^	n.d.	3.2 × 10^4^ ± 4.3 × 10^3^
Low pH (modified pH)	n.d.	n.d.	n.d.	n.d.	n.d.	n.d.
E						
High pH (native pH)	n.d.	n.d.	3.8 × 10^6^ ± 3.8 × 10^5^	*2.2 × 10^6^ ± 4.0 × 10^5^	n.d.	n.d.
Low pH (modified pH)	n.d.	n.d.	n.d.	*1.8 × 10^5^ ± 2.8 × 10^4^	n.d.	n.d.
RR						
High pH (native pH)	*1.2 × 10^7^ ± 6.0 × 10^5^	*1.3 × 10^7^ ± 8.3 × 10^5^	4.3 × 10^5^ ± 9.5 × 10^4^	*1.2 × 10^6^ ± 7.9 × 10^4^	n.d.	1.1 × 10^4^ ± 3.7 × 10^3^
Low pH (modified pH)	*4.1 × 10^6^ ± 4.6 × 10^5^	*4.6 × 10^6^ ± 9.6 × 10^5^	n.d.	*3.4 × 10^5^ ± 8.5 × 10^4^	n.d.	n.d.
OP2						
High pH (modified pH)	n.d.	n.d.	2.7 × 10^6^ ± 3.4 × 10^5^	*1.7 × 10^6^ ± 1.5 × 10^5^	*4.6 × 10^4^ ± 7.8 × 10^3^	6.1 × 10^4^ ± 7.2 × 10^3^
Low pH (native pH)	n.d.	n.d.	n.d.	*2.5 × 10^5^ ± 3.5 × 10^4^	*2.0 × 10^4^ ± 5.9 × 10^3^	4.9 × 10^4^ ± 7.5 × 10^3^
OP7						
High pH (modified pH)	7.7 × 10^7^ ± 3.2 × 10^6^	*7.0 × 10^7^ ± 1.0 × 10^7^	*1.9 × 10^5^ ± 2.1 × 10^4^	*1.6 × 10^5^ ± 5.3 × 10^4^	n.d.	3.4 × 10^4^ ± 6.7 × 10^3^
Low pH (native pH)	1.7 × 10^7^ ± 1.7 × 10^6^	*1.4 × 10^7^ ± 3.5 × 10^5^	*3.4 × 10^4^ ± 8.6 × 10^3^	*4.0 × 10^4^ ± 5.2 × 10^3^	n.d.	3.3 × 10^4^ ± 2.0 × 10^4^

a Abundance of growing organisms was estimated either as the temporal increase in abundance of total AOA, AOB, and comammox *amoA* genes after incubation for 30 days (i.e., final abundance – initial abundance) (by qPCR) or as the abundance of ^13^C-labeled *amoA* genes in the heavy fraction (by SIP). n.d., no detectable growth; *, significant difference (*P < *0.05) between low pH and high pH for each soil (*t* test statistics).

10.1128/mSystems.00309-20.2 FIG S2 Comparison of AOA, AOB, and comammox growth levels estimated by temporal increase of total *amoA* gene abundance after incubation for 30 days (by qPCR) or by the abundance of ^13^C-labeled *amoA* genes in the heavy fractions (by DNA-SIP). The *x*-axis and *y*-axis data were set to the same range of values, with the dotted line representing the same values assessed by both approaches. The area above the dotted line indicates higher growth estimation by SIP than qPCR, while the area below the line indicates higher growth estimation by qPCR than SIP. Each unfilled circle represents ammonia oxidizer growth from a single soil microcosm. Soil microcosms with no growth of a specific ammonia oxidizer group detected by both approaches are not displayed in the plot. Download FIG S2, TIF file, 0.1 MB. Copyright © 2020 Zhao et al. 2020 Zhao et al. This content is distributed under the terms of the Creative Commons Attribution 4.0 International license.

Putative contributions of autotrophic ammonia oxidizers to nitrification, which were estimated according to the number of autotrophic (^13^C-labeled) ammonia oxidizers, indicated that either AOA or AOB dominated net nitrification depending on the soil type, with little influence of soil pH perturbation (Fig. 1b). AOB dominated ammonia oxidation in the two forest soils and the young oil palm soil, while AOA were the dominant ammonia oxidizers in the long-established oil palm soil. The riparian soil was the only soil in which both AOA and AOB contributed similarly to nitrification in both native and pH-modified soils (Fig. 1b). Comammox activity contributed very slightly to nitrification (Fig. 1b). Although pH modification never inverted the relative contributions of AOA and AOB to the net NO_x_^−^ production, AOB growth was inhibited following acidification of one forest soil (LF).

A relatively large proportion of NO_x_^−^ production was unexplained in two native-pH soils (forest LF and young oil palm OP2) as shown by estimations of contributions analyzed either by the number of autotrophic (^13^C-labeled) ammonia oxidizers (Fig. 1b) or by the temporal increases in total ammonia oxidizer abundances (Fig. S3), based on current knowledge of cell-specific activities of AOA, AOB, and comammox. This indicates either other potential sources of ammonia oxidation in these two soils or higher ammonia-oxidizer-specific cell activity than previously recorded. In addition, the possibility of underestimation of AOB contributions in the native LF soil due to decreases in AOB abundance during the later stages of incubation (Fig. 2) that were in turn due to low availability of ammonium (Fig. S1) cannot be ruled out.

10.1128/mSystems.00309-20.3 FIG S3 Data were determined as described for Fig. 1b in the main paper, except for the estimated contributions of ammonia oxidizers. In this figure, the relative contributions of AOA, AOB, and comammox to the NO_x_^−^ production were estimated as their temporal increase in *amoA* gene abundance during incubation (final abundance − initial abundance) and their recorded highest maximum specific cell activity (2.6 fmol NH_3_ cell^−1^ h^−1^ for AOA, 23 fmol NH_3_ cell^−1^ h^−1^ for AOB, and 2.6 fmol NH_3_ cell^−1^ h^−1^ for comammox). Download FIG S3, TIF file, 0.1 MB. Copyright © 2020 Zhao et al. 2020 Zhao et al. This content is distributed under the terms of the Creative Commons Attribution 4.0 International license.

## DISCUSSION

### Differential stabilities of ammonia-oxidizing communities in response to pH perturbation.

In this study, stability was defined as the combined effects of the immediate response to disturbance (resistance) and the following recovery over time (resilience) (33). Stability data, estimated as the proportional change in archaeal, bacterial, or comammox *amoA* gene abundances after pH perturbation, were used to address the impact of pH perturbation on different AO populations. This calculation potentially suffers from a bias in the interpretation of stability as a consequence of inclusion of the total ammonia oxidizer abundance (estimated by qPCR) (Fig. 3) rather than the ^13^C-labeled ammonia oxidizer abundance (estimated by DNA-SIP) (see Fig. S4 in the supplemental material) to evaluate AO stability in response to pH perturbation. Potentially dormant and nonactive ammonia oxidizers can be included in the qPCR-based calculation, which might undermine interpretation of the stability of a microbial group in response to a pH perturbation. However, in contrast, the DNA-SIP approach might be too restrictive, given our limited knowledge of the physiology of terrestrial ammonia oxidizers, especially AOA and comammox (38, 39). Indeed, DNA-SIP estimates growth of autotrophic but not heterotrophic (or mixotrophic) organisms (40), and some active but nonreplicating cells might also be ignored by this approach (41). In addition, the aim of our study was to compare the stabilities of the different AO groups following soil pH perturbation but the three AO groups were often growing preferentially under different conditions (Fig. 1b), possibly due to environmental selection or competition (37). Therefore, the study compared the impacts of pH perturbation on different ammonia oxidizer groups at the whole-community level, rather than investigating only the active groups revealed by DNA-SIP.

10.1128/mSystems.00309-20.4 FIG S4 The proportional change of ^13^C-labeled AOA, AOB, and comammox abundance following pH modification (acidification or liming) and incubation for 30 days of the five soils from the land-use the gradient: two forest soils (LF and E), a riparian soil (RR) and 2- and 7-year-old oil palm soils (OP2 and OP7). ^13^C-labeled AOA in soils LF, E, and RR and ^13^C-labeled comammox in soil E were not detected by DNA-SIP and thus are not included in the plot. The asterisk above or below a pair of bars indicates a significant difference between proportional changes of AOA, AOB, or comammox in the corresponding soil (*P* < 0.05). Download FIG S4, TIF file, 0.09 MB. Copyright © 2020 Zhao et al. 2020 Zhao et al. This content is distributed under the terms of the Creative Commons Attribution 4.0 International license.

The present report provides evidence for greater stability of AOA than of AOB in response to short-term pH perturbations. Higher stability is indicated by a small deviation from the neutral point (stability = 0), reflecting the fact that abundances in the perturbed and native soils were similar. The changes in AOA abundance following pH modification were smaller than those in AOB in all soils except riparian soil, where AOA and AOB abundances were affected to the same extent by soil acidification. The higher stability of AOA might result from greater tolerance of (resistance to) pH changes, as previously observed (16), or from faster recovery (resilience) of ammonia oxidation activity after pH modification. The isolation of both acidophilic (e.g., “*Candidatus* Nitrosotalea devanaterra” [42]) and neutrophilic (e.g., Nitrososphaera viennensis [43] or “*Candidatus* Nitrosocosmicus franklandus” [44]) AOA demonstrates that physiologically distinct but potentially redundant AOA clusters exist in soil. The growth of functionally redundant AOA populations following changes in soil pH would facilitate maintenance of or even increased ecosystem function activity (45). This is especially true for ammonia oxidation activity as ammonia oxidizers have previously been shown to adapt rapidly after environmental perturbations such as drought-rewetting (34, 35, 46) or copper application (47, 48). However, the present study was unable to determine which mechanism(s) (resistance, resilience, or redundancy) is more important in providing higher stability to the AOA populations, as investigation of these mechanisms would require more-frequent monitoring of community composition.

AOB abundance was more affected by pH changes than AOA abundance (except in one soil), with a substantial decrease occurring following soil acidification, while the abundances of both AOA and AOB increased following soil liming. A temporal increase of ammonia oxidizer abundance is often linked to nitrification activity in soil (8, 20–22, 31, 49–51). Our study results support previous suggestions of AOB abundance as a reliable biotic indicator of multiple soil functions, including N cycling (52, 53). Similarly, autotrophic AOB activity (estimated as ^13^C-labeled AOB growth) in all soils was affected by pH changes, but this was not always the case for AOA or comammox (Table 1). These results collectively imply that AOB are good bioindicators of the consequences associated with soil nitrification following pH perturbations resulting from conversion of tropical forests to oil palm plantations.

The comammox species displayed higher stability than AOA and AOB. For instance, in the young oil palm soil, comammox abundance was less affected by liming than AOA and AOB abundance (Fig. 2). Additionally, in two logged forest soils, comammox abundance was not affected by acidification, while both the AOA and AOB populations suffered from undercompensation (Fig. 2). Therefore, comammox seem to be more stable than AOA or AOB in response to a short-term pH perturbation, but because the presence and activity of comammox in different pH soils are much less extensively documented than those of AOA and AOB, further investigation in terrestrial ecosystems is required to test this hypothesis.

### Growth and activity of ammonia oxidizers in acidic soils.

Acidophilic or acidotolerant nitrifiers are essential for maintenance of nitrification in low-pH environments, and several obligate acidophilic AOA have been isolated (42, 54). These AOA likely play a dominant role in ammonia oxidation in acidic soils (18–20), although their mechanisms for adaptation to acidic conditions are still not fully resolved (55). Despite the frequent presence of AOB phylotypes in acidic soils (16, 36, 56–59), their contribution to ammonia oxidation activity in low-pH soils is considered to be low and can be explained by urease activity (24). The present study results confirmed the activity of AOA under acidic conditions but also provide evidence for AOB activity in several acidic-pH soils. Indeed, autotrophic ammonia oxidation was largely attributed to the presence of betaproteobacterial AOB in three acidic soils (i.e., young oil palm, forest E, and riparian soils, all at pH < 5.0 throughout incubation) (Fig. 1 and 4). These results indicate that at least some of the *Nitrosospira* or *Nitrosomonas* phylotypes can physiologically adapt to low pH and contribute strongly to soil nitrification in some acidic or acidified tropical soils, as recently observed in acidic Scottish soils (37) and in fertilized acidic forest and rice paddy soils (60, 61), but the mechanism remains unknown. A study of *Nitrosospira* niche specialization in soils recently suggested that some abundant phylogenetic clades, currently uncultivated and without genome representatives, are ubiquitous in acidic soils (17). It is interesting that their activity and growth may have been limited by ammonium availability in two of the low-pH soils (LF and OP2 soils) (Fig. S1). The presence of AOA without growth or [^13^C]CO_2_ incorporation in those soils (Table 1) also suggests that competition for ammonia between AOB and AOA might have occurred, which is consistent with previous observations of similar ammonia affinities of several AOB and AOA in cultures (62) and in soil (29, 37). Therefore, the present study expands our knowledge of the pH adaptation range of AOB in soils.

Surprisingly, DNA-SIP provided further evidence of autotrophic growth of comammox in the oil palm soils even under acidic condition (Fig. 4), although the estimated contribution to nitrification was low (Fig. 1). All currently cultivated comammox strains were isolated from aquatic systems, which grow preferentially under neutral to slightly alkaline conditions (pH 7.0 to 7.8) (63, 64), while little is known of the activity of comammox in terrestrial ecosystems or under low-pH conditions. The present study provided new and robust evidence for nitrification that was attributed to acid-tolerant or acidophilic comammox in soil. In a preliminary investigation of comammox in the soil, primers specifically targeting either clade A or clade B comammox *amoA* genes were tested and revealed that clade A but not clade B comammox organisms were present in the oil palm soils. Therefore, growth of clade A comammox organisms was likely detected by DNA-SIP in these soils, in contrast to recent findings in other soils (65). However, the numerous nonspecific amplification products obtained using the specific primers distinguishing clade A and clade B prevented their accurate use in a quantitative approach.

### Impact of land-use change on nitrification and associated microbial communities.

The riparian soil represented the environment least disturbed by human activity as this area was not being subjected to deliberate intensive anthropogenic exploitation such as logging or conversion to oil palm plantation. However, human activity at nearby sites may have inevitable influences on this area, such as fertilizer runoff from the oil palm plantations and sediment transported by erosion due to upstream logging activities. In contrast to other soils, nitrification activity and associated communities (in terms of AOA/AOB/comammox ratios) appeared unchanged after pH perturbation of the riparian soil, while the relative contributions of AOA and AOB to nitrification in this soil (with unique similar activities) also remained stable (Fig. 1b). Comammox activity was believed to be minimal or absent during incubation of the riparian soil, but monitoring of comammox abundance revealed undercompensation similar to that seen with AOA and AOB following soil acidification (Fig. 2). The diversified guild of active ammonia oxidizers might be of great importance in stabilizing ecosystem functioning through functional redundancy following environmental disturbance (66–69), as pH perturbation had a limited impact on ecosystem function in this soil (Fig. 1). However, community assessment would be required to fully assess this hypothesis. We therefore suggest that the intensified anthropogenic activities might have affected the diversity of nitrifiers in tropical soils, thereby negatively affecting the response to environmental disturbance of microbes involved in important steps of the nitrogen cycle.

A previous meta-analysis did not identify an overall impact of soil pH on soil net nitrification rates across a wide range of ecosystems (25), but pH perturbations of specific soils often change nitrification rates, with long-term liming resulting in increased nitrification rates (70–72). In the present study, despite growth of AOA and AOB over a range of soil pH values, abundance and activity, and resulting contributions to nitrification, were lower at low pH after soil acidification (except for the riparian soil) and showed a contrasting trend over a short period of time (i.e., before ammonia presumably became limited due to low soil mineralization) (Fig. 1 and 2). In commercial plantation fields, low mineralization rates occur naturally due to low understory vegetation (with the exception of accumulation of decaying palm leaves near the tree stems). However, the constant supply of ammonium fertilizer to the oil palm plantation soils in the form of “open fertilizer bags” prevents ammonia limitation and results in a low but persistent nitrification rate. This intensive ammonium-based fertilization associated with oil palm conversion induces a decrease in soil pH (7, 8) and in the nitrification rate and therefore enhances the nitrogen utilization efficiency of the fertilizers for the plants as the ammonium remains bounded to clays and other soil particles (73). This is also likely to reduce N_2_O production associated with ammonia oxidation, especially by AOB, which produce higher N_2_O yields than AOA (74). In contrast, in native tropical forest sites, continuous leaf fall induces high mineralization rates, preventing ammonia limitation. A year-round study estimated annual N_2_O emissions in oil palm fields of 1.2 kg N ha^−1^ (75). This would putatively contribute to a total of 0.02 Tg N_2_O-N per year, accounting for 0.6% of N_2_O emissions from croplands worldwide (76). Interestingly, it was observed that the unfertilized or moderately fertilized oil palm soils did not produce more N_2_O than primary forest soils (75, 77, 78), while intensive fertilization increased the total N_2_O emission 171-fold (77). The pH decline associated with land conversion might have restricted short-term N_2_O production associated with nitrification, but the N_2_O emission in oil palm fields inevitably rises following long periods of intensive N fertilization. Therefore, the quantitative environmental impact of conversion of tropical forests to commercial plantation fields, including that on the global nitrogen cycle and on greenhouse gas emission, needs to be fully considered and quantified to provide comprehensive recommendations for future land-use ecosystem conversions.

## MATERIALS AND METHODS

### Soil sites.

Soil was collected in November 2016 from rainforests of northeastern Borneo Island (Malaysia) (4°49′N, 116°54′E) within the sites of the Stability of Altered Forest Ecosystem (SAFE) Project (www.safeproject.net) (79). This is a long-term landscape-scale project used to study the effects of anthropogenic activity linked to deforestation and oil palm agriculture on tropical ecosystems. The soils at SAFE are classed as orthic Acrisols or Ultisols. All sample collection sites were situated within a 35-km^2^ region that includes two forest soils (LF and E) with a history of selective logging of dipterocarps, 2- and 7-year-old oil palm soils (OP2 and OP7), and a riparian soil (RR) nearby the OP7 field. One major difference between LF and E sites is that the former contains a lower proportion of pioneer tree species (80). Ammonium sulfate was applied at a rate of 2 kg N per palm tree in the form of fertilizer bags three times per year. Composite samples were collected for each soil type from the upper 10-cm surface soil layer (horizon A), and the pooled soil samples from each site were air-dried before transport at ambient temperature to the United Kingdom. Soils were rewetted and incubated at 25°C for 8 weeks to restore microbial activity and were then collected and stored at 4°C before construction of microcosms. Additional site and soil descriptions, including climate, canopy, and soil topography, were detailed in a previous study (80, 81) and some *in situ* soil characteristics are presented in Table S1 in the supplemental material.

10.1128/mSystems.00309-20.5 TABLE S1 Soil physiochemical characteristics measured *in situ*. Total soil C and N levels were measured from the upper 1-to-10-cm soil layer, and total litter C and N levels were measured from the leaf litter above the soil surface. For each site, characteristics means and standard deviations were determined with at least 4 biological replicates. *, OP7 had only one replicate with leaf litter coverage. Download Table S1, DOCX file, 0.02 MB. Copyright © 2020 Zhao et al. 2020 Zhao et al. This content is distributed under the terms of the Creative Commons Attribution 4.0 International license.

### Soil microcosms.

Triplicate microcosms were constructed with each soil either at its original pH or after modification of pH. For pH modifications, the pH of the 3 native neutral soils (LF, E, and RR; pH 5.9 to 6.8) was reduced to pH 4.5 by addition of Al_2_(SO_4_)_3_, while the pH of the two acidic oil palm soils (OP2 and OP7; both pH 5.0) was increased to pH 6.5 by addition of Ca(OH)_2_. Each microcosm contained 13 g of wet soil (30% [wt/wt]) water content) in a 120-ml serum bottle sealed with a butyl rubber stopper and an aluminum cap. Either isotopically labeled [^13^C]CO_2_ or [^12^C]CO_2_ gas was added to the headspace air of each bottle by replacement, to give a final concentration of 5% CO_2_ (vol/vol). All microcosms were incubated in the dark at 28°C for 30 days and were aerated, and resupplied with CO_2_, every 3 days, to ensure adequate O_2_ supply for nitrification and to avoid dilution of ^13^CO_2_ by ^12^CO_2_ gas originating from soil respiration. All microcosms were destructively sampled after incubation for 15 and 30 days, and the collected soils were frozen at –80°C.

### Measurement of soil nitrification and pH.

Ammonium (NH_4_^+^) and nitrite plus nitrate (NO_x_^−^) concentrations were determined colorimetrically as previously detailed (29). In brief, 2 g soil was mixed with 10 ml of 1 M KCl for 30 min and supernatant was collected after centrifugation at 3,000 × *g* for 15 min for assay of NH_4_^+^ and NO_x_^−^ concentrations. Soil pH was measured in a soil suspension/water mixture (1:2 [wt/wt]). These measurements were taken before (day 0), during (day 15), and after (day 30) incubation of the microcosms.

### DNA extraction and quantification of ammonia oxidizer abundance.

DNA was extracted from 0.5-g soil samples using a FastDNA spin kit for soil (MP Biomedicals, Santa Ana, CA, USA) following the manufacturer’s instructions. The quantity and quality of DNA extracts were assessed using a NanoDrop spectrophotometer (Thermo Fisher Scientific, Waltham, MA, USA). The abundance of the archaeal (AOA) and canonical bacterial (AOB) ammonia monooxygenase subunit A (*amoA*) gene was estimated by qPCR on total DNA extracts (diluted to 5 ng μl^−1^) using primer sets amoA23f/amoA616r (82) and amoA1F/amoA2R (83), respectively, and qPCRs were performed as described previously (74). In addition, complete ammonia oxidizer (comammox) *amoA* levels were quantified using the primer pair Ntsp-amoA 162F/359R targeting both the A and B comammox clades (84) in a 20-μl reaction mixture consisting of 10 μl iQ SYBR green supermix, 0.4 μg bovine serum albumin (BSA), and a 0.5 μM concentration (each) of the primers. Conditions of the qPCR cycles were as follows: 95°C for 5 min and 40 cycles of 95°C for 30 s, 58°C for 30 s, and 72°C for 1 min followed by measurement of fluorescence. The standards containing 10^1^ to 10^8^ genes per reaction mixture were used for comammox qPCR with a reaction mixture containing an equimolar mix of 23 of 40 sequenced clones amplified by Ntsp-amoA 162F/359R from the soils, to cover the degeneracy of the primers as much as possible. Amplification efficiencies for *amoA* gene quantification were in ranges of 83% to 87% for AOA, 91% to 99% for AOB, and 96% to 103% for comammox, with *R*^2^ values of >0.99. Amplification specificity was assessed by melting curve analysis and standard agarose gel electrophoresis. Primers (six forward or six reverse primers, respectively) were also tested using equimolar mixtures of oligonucleotides specifically targeting either clade A or clade B comammox *amoA* genes as described previously (85), but the resultant numerous nonspecific PCR products prevented the use of these primers for quantification of comammox in our soils.

### Stable-isotope probing.

Isopycnic density gradient centrifugation was performed on DNA extracted from each 30-day microcosm sample as previously described (22, 37, 86). Briefly, 1 μg DNA was mixed in 8.5 ml CsCl solution. The mixture was adjusted to a final CsCl buoyant density of 1.71 g ml^−1^ and then transferred to 8-ml quick-seal polyallomer tubes (Beckman Coulter, Palo Alto, CA, USA) before centrifugation in a MLN80 rotor (Beckman Coulter) was performed at 45,000 rpm for 60 h at 20°C. Each tube was divided into 15 fractions (500 μl each), polyethylene glycol was used to precipitate DNA, followed by 70% ethanol purification, and the resultant DNA pellet was dissolved in 30 μl sterile water. AOA, AOB, and comammox *amoA* gene abundances were then determined in each DNA fraction (fractions 2 to 14) by qPCR as described above. Autotrophic growth of ammonia oxidizer communities was determined by comparing [^12^C]CO_2_ and [^13^C]CO_2_ incorporation profiles, i.e., when the buoyant density peaks were distinct between the two treatments.

### Statistical analyses.

All statistical tests were performed in Statistics 23.0 (SPSS, Chicago, IL, USA). The net nitrification rate was defined as the increase of NO_x_^−^ after incubation for 30 days, and the increase was considered significant if it differed from the null hypothesis (no change) using a Student's *t* test. Two-way analysis of variance (ANOVA) was employed to assess the effect of soil type (gradient of forest to oil palm) and pH perturbation (acidification or liming) on net nitrification rate, followed by a Tukey *post hoc* test to determine significant differences in means. One-way ANOVA was employed to determine the effect of incubation time (days 0, 15, and 30) on soil pH, NO_x_^−^ and NH_4_^+^ concentrations, and ammonia oxidizer abundance.

The putative contributions of AOA, AOB, and comammox to nitrification were estimated by multiplying data representing their respective levels of growth by their maximum specific cell activities measured for pure cultures of AOA (for Nitrososphaera viennensis, 2.6 fmol NH_3_ cell^−1^ h^−1^) (87), AOB (for Nitrosospira multiformis, 23 fmol NH_3_ cell^−1^ h^−1^) (88) and comammox (for Nitrospira inopinata, 2.6 fmol NH_3_ cell^−1^ h^−1^) (62). The comammox cell-specific activity estimation is based on an estimated *V*_max_ of 14.8 μmol NH_3_ mg^−1^ protein h^−1^ (62), assuming a conversion factor of 5.7 g wet weight cell g^−1^ of protein (62) and that 1 g wet weight of bacteria usually contains around 10^12^ cells (estimated on the basis of previously reported Escherichia coli data [89]). N. viennensis and N. multiformis were used as they are representative soil ammonia oxidizers and have high cell-specific activity, while N. inopinata was used due to exclusive detection of clade A comammox by clade-specific primers. Ammonia oxidizer growth was estimated as the temporal increase in *amoA* gene abundance during incubation (i.e., final abundance – initial abundance) or as the number of cells assimilating CO_2_, estimated by the number of cells in the heavy fractions of the [^13^C]CO_2_-labeled microcosms, and both approaches were used to estimate putative contributions of AOA, AOB, and comammox to nitrification.

The stability index was calculated to reflect the degree of variation in ammonia oxidizer abundances following a 30-day pH perturbation. It was proposed that ecosystem stability comprises two components: resistance and resilience. Since our sampling frequency was not sufficient to allow us to monitor and distinguish resistance (immediate response to disturbance) and resilience (recovery over time) phases as defined by Griffiths and Philippot (33), stability (representing the combined effects of resistance and resilience) was used to describe and compare the impacts of pH perturbation on different AO populations in this study. The stability of ammonia oxidizer community abundance following a pH modification (acidification or liming) was estimated by calculating the proportional change in archaeal, bacterial, or comammox *amoA* gene abundances in modified-pH (*M*) soil compared to native-pH (*N*) soil at day 30, using the following equation: $\text{stability }=\frac{\left(M-N\right)}{N}\times 100$ (90). Stability values can therefore range from a minimum negative value (−100%) to an unbounded maximum positive value, and the sign and deviation from the neutral point (stability = 0, indicating no change compared to unperturbed control) are used to interpret the magnitude of the compensation mechanisms following perturbation. Indeed, perturbation might change the community activity but trade-off of compensatory mechanisms (such as functional redundancy, resistance, and/or resilience) would stabilize the community activity at a novel threshold that would be either lower or higher than that seen with the unperturbed community. Therefore, negative and positive ecosystem function stability values indicate under- and overcompensation following perturbation, respectively. Stability was expressed as a proportional rather than an absolute change to allow comparisons between different soils, and independent Student’s *t* tests were used to compare the stability indices of AOA, AOB, and comammox communities in each soil and to determine if the perturbation induced similar compensation mechanisms for these ammonia oxidizer communities. Additionally, proportional changes in ^13^C-labeled ammonia oxidizer abundance after pH perturbation were calculated using the same formula.
