# Supplementary material for: Differential Ecosystem Function Stability of Ammonia-Oxidizing Archaea and Bacteria following Short-Term Environmental Perturbation
Source: mSystems. 2020 Jun 16;5(3):e00309-20. doi: 10.1128/mSystems.00309-20 (PMC7300361; doi:10.1128/mSystems.00309-20)
Supplement: TABLE S1 [file mSystems.00309-20-st001.docx]

|  | bulk density  (g cm^-3^) | total soil N  (g kg^-1^) | total soil C  (g kg^-1^) | C/N | total litter N  (g kg^-1^) | total litter C  (g kg^-1^) |
| --- | --- | --- | --- | --- | --- | --- |
| LF | 0.80 ± 0.16 | 2.4 ± 1.4 | 32.1 ± 20.4 | 144.1 ± 49.7 | 17.6 ± 3.9 | 364.4 ± 68.2 |
| E | 0.84 ± 0.21 | 3.8 ± 2.6 | 64.0 ± 67.2 | 138.3 ± 54.4 | 18.2 ± 1.5 | 400.1 ± 38.8 |
| RR | 1.25 ± 0.10 | 1.4 ± 0.6 | 11.8 ± 3.2 | 96.2 ± 36.1 | 17.8 ± 2.8 | 406.2 ± 58.8 |
| OP2 | 1.22 ± 0.12 | 0.5 ± 0.2 | 7.0 ± 2.1 | 139.7 ± 18.1 | 17.0 ± 2.3 | 396.1 ± 82.5 |
| OP7 | 1.28 ± 0.18 | 0.7 ± 0.5 | 9.7 ± 4.7 | 152.4 ± 41.8 | 15.4* | 319.9* |
